# The Effect of Omega-3 Fatty Acid Supplementation on Maternal Depression during Pregnancy: A Double Blind Randomized Controlled Clinical Trial

**Published:** 2014-07

**Authors:** Maasumeh Kaviani, Laleh Saniee, Sara Azima, Farkhondeh Sharif, Mehrab Sayadi

**Affiliations:** 1 Department of Midwifery, School of Nursing and Midwifery, Shiraz University of Medical Sciences, Shiraz, Iran;; 2 Student Research Committee, Shiraz University of Medical Sciences, Shiraz, Iran;; 3 Community Based Psychiatric Care Research Center, Department of Midwifery, School of Nursing and Midwifery, Shiraz University of Medical Sciences, Shiraz, Iran;; 4 Community Based Psychiatric Care Research Center, Department of Mental Health and Psychiatric Nursing, School of Nursing and Midwifery, Shiraz University of Medical Sciences, Shiraz, Iran; 5 Department of Biostatistics, School of Public Health , Behbahan University of Medical Sciences, Behbahan, Iran

**Keywords:** Depression, Omega-3 Fatty Acid, Pregnancy

## Abstract

**Background: **Depression is one of the most debilitating disorders during pregnancy and its recovery and treatment are among the concerns of obstetrics and gynecology experts.

The present study aimed to determine the effect of omega-3 supplement on mild depression during pregnancy among primiparous women.

**Method: **In this double-blind clinical trial, 80 primiparous women were randomly divided into 2 groups of omega-3 and placebo. The experimental group received 1 g omega-3 capsules for 6 weeks. The study data were collected by completing Beck Depression Inventory before and 6 weeks after the intervention.

**Results: **The results revealed a significant difference between the two groups regarding the mean score of depression before and after the intervention (P<0.001). Besides, the mean difference of depression score before and after the intervention was significantly higher in the omega-3 group (P<0.001).

**Conclusion: **Considering the study results, using omega-3 supplement is a suitable method for recovery from mild depression during pregnancy with no complications for mothers and infants.

**Trial Registration Number: **IRCT2012121011717

## Introduction

Depression is one of the most prevalent and debilitating psychiatric disorders which puts heavy economic and social expenses on the society.^1^ Pregnancy is a stressful period which is accompanied by physiological and mental changes. Some women also suffer from depression during this period.^2^

Studies have shown that depression during pregnancy may disrupt fetal growth. Mother’s stress during pregnancy is also accompanied by low birth weight, premature birth, and fetal h*ypoxia.^3^* To recover from depression in pregnancy, drug treatment and psychological consultations are used. Prescribing Tricyclic antidepressants is a prevalent treatment for depression; however, consuming medicine has some limitations during pregnancy.^4^^,^^5^ Additionally, many mothers refuse taking antidepressants due to being afraid of their side effects on the fetus.^6^ In the recent decade, non-pharmacological treatments and those with fewer complications have been considered for controlling some psychiatric syndromes.^7^^-^^10^ Use of unsaturated fatty acids for treatment of depression has also been reported in some studies.^11^ Evidence has shown the effect of omega-3 on serotonin of cerebrospinal fluid and a relationship between reduction of its level in the cellular membrane and plasma of the depressed patients.^12^

Reduction of omega-3 fatty acid has a considerable effect on development of depressive moods, negative attitude toward life, and committing suicidal behaviors.^8^ Since it is necessary and inevitable to search for alternative methods with fewer complications for mothers and fetuses, this study aims to determine the effect of omega-3 fatty acid on mild depression during pregnancy in primiparous women.

## Materials and Methods

**Figure 1:**
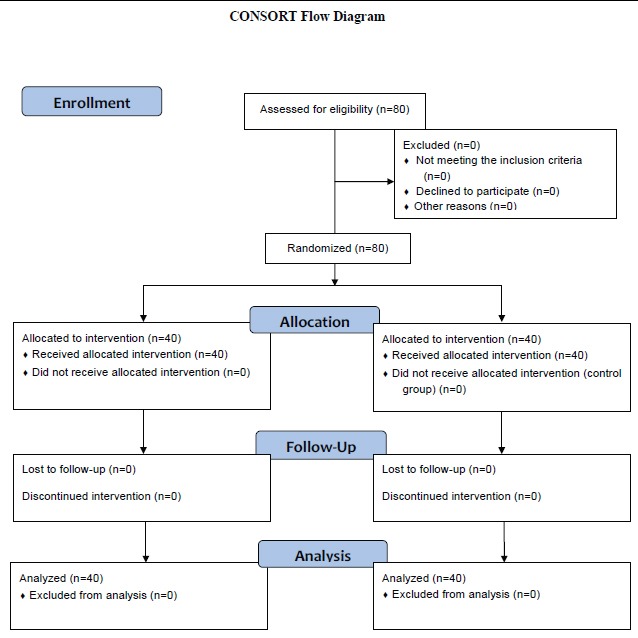
CONSORT flow diagram of the participants

The present double blind randomized controlled clinical trial was conducted on 80 primiparous women suffering from mild depression who had referred to Shiraz health centers. This study was performed from September 2012 to March 2012. Among all the health centers, two centers were selected using simple random sampling and 40 individuals from each center were enrolled into the study. The participants were selected through systematic sampling and were assigned to an intervention and a control group using permuted block randomization. A table of random numbers was also used to determine which of two pairs of samples could be located in one group. The protocol of the study has been shown in figure 1.

Depression was assessed using Beck Depression Inventory (BDI). BDI is the most widely-used instrument for self-assessment of depression in clinical research. It consists of 21 items, each having four response options. The scale is utilized to rate the severity of depression in individuals aging 13 years or above.

Beck et al. (1996) obtained the test-retest reliability coefficient of BDI to be 93% in a one-week interval.^13^

The reliability of BDI has been reported to vary between 0.7 and 0.9 in different studies.^13^ After filling out Beck’s questionnaire, if a score of 14-19 was obtained, demographic information checklist was also completed. One week after referring to the psychiatrist, in case their mild depression and no need for drug treatment were confirmed, people were included in the study after receiving a letter of consent.

Omega-3 fatty acid (1 g per day for 6 weeks) and placebo (containing olive oil) (1 g per day for 6 weeks) were given to the study participants. Both  mothers and researcher  were blinded to drug and placebo. The samples were studied at the end of each week regarding the intensity of pregnancy depression, monitoring the intake of capsules, including monitoring the regular daily intake of capsules and emergence of potential complications. BDI was completed again after 6 weeks of intervention*.*

The inclusion criteria of the study were being primiparous with intrauterine pregnancy over 20 weeks, obtaining a score of 14 to 19 in BDI, being above 18 years old, not consuming fish twice a week (those who regularly used fish were removed from the study and were replaced by the next individual), not suffering from schizophrenia, bipolar disorders, blood disorders, such as VonWillebrand, hypertension, and hyperlipidemia, and renal and thyroid diseases, not taking anticoagulants and antidepressants, not smoking or using narcotics, and not participating in activities such as yoga, relaxation, and psychological consultations. On the other hand, the exclusion criteria of the study were having allergic reaction or digestive disease to the medicines. 

This clinical trial received its permit from the Ethic Committee of Shiraz University of Medical Sciences (code: 91-6111) and clinical trial center. Besides, written informed consents were obtained from all the study participants. After all, the data were entered into the SPSS statistical software (v. 16) and analyzed using descriptive statistics and paired and independent t-test. Additionally, P<0.05 was considered as statistically significant.

## Results

This study was conducted on 80 pregnant women who had referred to Shiraz health centers. The results revealed no significant difference among the participants regarding the demographic characteristics (table 1).

**Table 1:** Demographic variables of the two groups

**Variable**	**Omega 3**	**Placebo**	**P value**
Age (mean±SD)	26.33±4.2	25.15±4.2	0.217
Education			
<6 years	3 (7.5%)	3 (7.5%)	0.615
6 to 9 years	5 (12.5%)	6 (15%)	
9 to 12 years	8 (20%)	4 (10%)	
High school	9 (22.5%)	15 (37.5%)	
Academic	15 (37.5%)	12 (30%)	
Occupation			
Homemaker	37 (92.5%)	40 (100%)	0.210
Employee	2 (5%)		
Other jobs	1 (2.5%)		

Comparison of the mean score of depression score before and after the intervention showed a statistically significant difference in the omega-3 group (P<0.001) (table2). The results showed that the depression score decreased by 9.17±5.3 in the omega-3 group and by 14.7±6.46 in the placebo group. Moreover, a statistically significant difference was observed between the two groups in this regard after the intervention (P<0.001) (table 2).

**Table 2:** Comparison of the mean score of depression before and after the intervention in the two groups

**Group**	**Mean score of depression before the intervention**	**Mean score of depression after the intervention**	**Mean difference**	**P value**
Omega 3	16.52±3.1	9.17±5.3	-7.35±6.18	<0.001
Control	17.47±4.05	14.7±6.46	-2.7±7.2	<0.021
P value	0.256	<0.001		

## Discussion

The present study aimed to investigate the effect of omega-3 capsule on mild depression of pregnancy among primiparous women. The study results showed a significant decrease in the mean depression score in the omega-3 group. These results were in line with those obtained by Saki et al. (2010) regarding the effect of omega-3 on treatment of depression. However, Saki et al. found no significant difference in the mean depression scores and performance levels of the two groups treated with omega-3 and Nortriptyline after 6 weeks of intervention. This finding showed that omega-3, similar to Nortriptyline (one of the Tricyclic antidepressants), was effective in reduction of mean depression score, reduction of depression symptoms, and improvement of patients’ performance.^14^

Moreover, a research was conducted on 28 patients with major depression in two groups treated with omega-3 and placebo. The results of that study demonstrated that the patients in the omega-3 group had significantly lower depression scores.^15^ In another study, depression scores decreased in the pregnant women receiving omega-3.^16^ Similarly, pilot studies were performed in which omega-3 considerably reduced the symptoms of depression in the children between 6 and 13 years old and improved their performance.^17^^,^^18^ A considerable decrease in depression scores has been reported in the subjects receiving omega-3 supplement.^19^ Besides, Logan found a strong relationship between major depression and reduction of omega-3 fatty acids.^20^ Also, another study was conducted on effect of Eicosapentanoic Acid (EPA) on depression and revealed a significant difference between the EPA and the placebo group in terms of Beck’s mean depression score. EPA also resulted in a more desirable recovery over time. This study showed that EPA supplement was effective in improvement of depression and could be used as a supplement along with antidepressants for treatment of mild to medium depression.^21^ Ivan Bagha et al. compared the effectiveness of omega-3 fatty acids and placebo in treatment of mild to medium postpartum depression. The findings of that study indicated that consumption of 1 gr omega-3 for 8 weeks reduced postpartum depression.^22^

Evidence has shown a significant relationship between abnormal mechanism of fatty acids and depression, such a way that reduction of omega-3 fatty acids has a considerable effect on creation of depressed mood, development of negative attitude toward life, and commitment of suicidal behaviors.^9^^-^^12^

The main mechanism of effect of omega-3 on the nervous system is via affecting the phospholipids of nervous cells’ walls and correct performance as well as proper secretion of neurotransmitters. The second mechanism is via reducing cytokines. Cytokines reduce access to progenitor neurotransmitters and their metabolism and, consequently, reduce the function of hypothalamus, pituitary gland, and nervous system. Reduction of cytokines also helps increase Brain Derived Neurotrophic Factor (BDNF) polypeptide which is effective in growth and survival of nervous cells during growth and development. Increase of this polypeptide has a direct relationship with reduction of depression symptoms.^23^

In contrast, Hakkarainen et al. showed no associations between the dietary intake of omega-3 fatty acids or fish consumption and depressed mood, major depressive episodes, and suicide.^24^ Also, the findings of the study by other researchers demonstrated no significant relationship between fish and n-3 polyunsaturated fatty acid intake and postpartum depression.^25^

The results of another studies also showed no benefit (but also no evidence of harm) of a daily EPA+DHA supplement for mood disorders. Yet, pufa intake affected these disorders by increasing n-3 long-chain.^26^^,^^27^

One of the limitations of this study was that the pregnant women refused to receive drug during pregnancy, which was somewhat solved through consultation and training. In addition, because they might forget consuming omega-3, it was necessary to follow them up by regularly calling them.

## Conclusion

Considering results of this study, it seems that omega-3 supplement, at least for a short period of time, is a suitable supplement with no complications for treatment of mild depression during pregnancy. Yet, further studies with larger sample sizes are recommended to be conducted on medium to severe depression along with taking antidepressants.
